# Influence of T-2 and HT-2 Toxin on the Blood-Brain Barrier In Vitro: New Experimental Hints for Neurotoxic Effects

**DOI:** 10.1371/journal.pone.0060484

**Published:** 2013-03-27

**Authors:** Maria Weidner, Sabine Hüwel, Franziska Ebert, Tanja Schwerdtle, Hans-Joachim Galla, Hans-Ulrich Humpf

**Affiliations:** 1 Institute of Food Chemistry, Westfälische Wilhelms-Universität Münster, Münster, Germany; 2 Institute of Biochemistry, Westfälische Wilhelms-Universität Münster, Münster, Germany; University of Iowa, United States of America

## Abstract

The trichothecene mycotoxin T-2 toxin is a common contaminant of food and feed and is also present in processed cereal derived products. Cytotoxic effects of T-2 toxin and its main metabolite HT-2 toxin are already well described with apoptosis being a major mechanism of action. However, effects on the central nervous system were until now only reported rarely. In this study we investigated the effects of T-2 and HT-2 toxin on the blood-brain barrier (BBB) *in vitro*. Besides strong cytotoxic effects on the BBB as determined by the CCK-8 assay, impairment of the barrier function starting at low nanomolar concentrations were observed for T-2 toxin. HT-2 toxin, however, caused barrier disruption at higher concentrations compared to T-2 toxin. Further, the influence on the tight junction protein occludin was studied and permeability of both toxins across the BBB was detected when applied from the apical (blood) or the basolateral (brain) side respectively. These results clearly indicate the ability of both toxins to enter the brain via the BBB.

## Introduction

**Figure 1:**
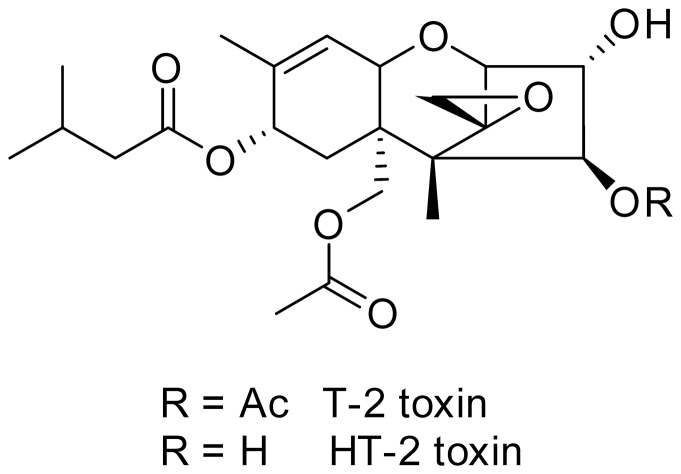
Chemical structure of T-2 and HT-2 toxin.

Mycotoxins are secondary fungal metabolites which are of importance for agricultural, ecological and toxicological reasons since they can cause economic losses due to contaminated cereals [1] and at the same time might be harmful for humans and animals due to their toxicological properties. Trichothecenes are a class of mycotoxins which is further divided into subgroups with T-2 and HT-2 toxin (Fig. 1) being the main representatives of the type A subgroup predominantly produced by fungi of the *Fusarium* species (*F. sporotrichioides* and *F. poae*). Chemically, trichothecenes are characterized by their sesquiterpenoid ring structure and the epoxide function between C12 and C13, which is crucial for the toxicological activity [2], [3]. Many cereal grains like wheat, corn, barley and oats are susceptible to T-2 toxin contamination and numerous studies detected T-2 as well as HT-2 toxin in different agricultural commodities [4]–[7]. Besides the co-occurrence of HT-2 toxin in T-2 toxin contaminated grains, HT-2 toxin is furthermore described as the main metabolite after T-2 toxin application in different *in vivo* as well as *in vitro* studies [3], [8]. For this reason the panel on contaminants in the food chain of the European Food Safety Authority has set the tolerable daily intake for the sum of both, T-2 and HT-2 toxin, at 100 ng/kg body weight [9].

Concerning the toxicity, T-2 and HT-2 toxin exhibit similar toxic properties: caspase-3-activation and subG1 formation as indicators of apoptosis were observed in primary human kidney cells for both toxins in similar concentration ranges [10], 11. Moreover, T-2 toxin is a well described inhibitor of eukaryotic protein synthesis. It reduces DNA and RNA synthesis and induces apoptosis in different tissues and cell culture models [3], . Additionally, acute toxic effects like vomiting, leukocytosis, skin irritation, hemorrhage and immune modulation are described in laboratory animals after T-2 toxin exposure [15], [16]. Besides many reports concerning the toxicity caused by T-2 toxin, toxic effects on the central nervous system (CNS), however, did not receive much attention [17] and neurotoxicity was regarded as not being one of the most critical effects of T-2 toxin by the European Food Safety Authority in 2011 [9]. Nevertheless, there have been reports of T-2 toxin induced effects on the CNS. Changes in levels of brain monoamines in certain areas of the rat brain after T-2 toxin application have been described [18] and oral administration of T-2 toxin in rats resulted in reduced motor activity and performance in a passive avoidance test [19]. Another *in vivo* study detected oxidative damage in brain of mice after per- and subcutaneous exposure to T-2 toxin [17]. Alteration of the blood-brain barrier (BBB) permeability was reported after subcutaneous application of T-2 toxin in mice which was mediated through oxidative stress [20] and changes in the amino acid permeability across the BBB have also been described [21].

The BBB is a selective barrier system within all organisms that exhibit a well-developed CNS [22]. It is formed by endothelial cells lining the cerebral microvessels and is responsible for regulating the brain microenvironment by restricting the transfer of different substances between blood and brain. Complex tight junctions between adjacent endothelial cells form the most important characteristic of the BBB as they limit the paracellular diffusion [22], [23].

Recent results from our group investigating the cytotoxic properties of T-2 toxin on primary human astrocytes showed that T-2 toxin triggered apoptotic reactions (caspase-3-activation) after short incubation times and was further rapidly metabolized to HT-2 toxin [24]. Astrocytes are important for the BBB as they form a complex network around the blood capillaries which is crucial for the maintenance of barrier properties [22]. Therefore, the question if T-2 toxin is able to cross the BBB to unfold its cytotoxic properties against astrocytes emerged.

The aim of this work was to examine the effects of T-2 and HT-2 toxin on the BBB *in vitro*. Both toxins were chosen since most studies concerning effects on the CNS dealt only with T-2 toxin neglecting its main metabolite HT-2 toxin which was already shown to exhibit similar cytotoxic effects as the parent toxin on various cell types [10]. HT-2 toxin was also formed following T-2 toxin application after short incubation periods in *in vitro* studies in primary human kidney cells [10], [11] as well as in a model reflecting the metabolism in the human gastrointestinal tract [25] indicating its potential presence *in vivo* after T-2 toxin ingestion. In this study, the BBB was mimicked by porcine brain capillary endothelial cells (PBCEC) seeded on polycarbonate Transwell® filter inserts [26]. The model can be related to the barrier function *in vivo* by measuring the transendothelial electrical resistance (TEER) which averages at about 1000 Ω*cm^2^ *in vitro* in comparison to an estimated TEER of 1800 Ω*cm^2^ *in vivo* [27]. Supplementation with physiological concentrations of hydrocortisone in the concurrent absence of serum is essential for the formation of barrier integrity in the used model where serum-free conditions allow improved reproducibility of experiments and permeability studies [28]. This model was already applied successfully in our group to study the effects of ergot alkaloid mycotoxins on the BBB [29]. In the present study, the general cytotoxicity of T-2 and HT-2 toxin on cells of the BBB (PBCEC) was evaluated by the CCK-8 assay which revealed strong cytotoxic effects of both toxins in low nanomolar ranges. Moreover, impairment of the barrier function was detected in a time dependent manner for T-2 and HT-2 toxin but in different concentration ranges. Additionally, incubation with toxins resulted in a loss of the tight junction protein occludin as detected by immunocytochemical staining and western blot analysis. Since effects on the BBB were observed for very low concentrations of T-2 and HT-2 toxin, a sensitive analytical method had to be developed using LC-MS/MS as a powerful technique to analyze nanomolar concentrations of both toxins in cell culture medium. Permeability across the BBB with accumulation of T-2 toxin on the basolateral side (resembling the brain side *in vivo*) was analyzed by LC-MS/MS with permeation of HT-2 toxin however, not being as pronounced as for T-2 toxin.

## Materials and Methods

### Chemicals

Both trichothecene mycotoxins, T-2 and HT-2 toxin, were biosynthetically prepared and isolated in our laboratory [30]. All chemicals including cell culture medium and supplements were purchased from Merck (Darmstadt, Germany), Sigma-Aldrich (Steinheim, Germany) or Biochrom AG (Berlin, Germany).

### Primary Cultures of Porcine Brain Capillary Endothelial Cells (PBCEC)

Primary cultures of porcine brain capillary endothelial cells (PBCEC) were isolated according to literature [26], [28]. The cells were prepared from brain tissue of freshly slaughtered pigs via enzymatic digestion and centrifugation steps before they were cryopreserved. For experiments, cryopreserved cells were prepared as described in recent reports [31], [32]. Briefly, PBCEC were thawed quickly at 37°C and seeded (250,000 cells/well) on rat tail collagen (0.54 mg/mL) coated 12-well Transwell® filter inserts with microporous polycarbonate membranes (Corning, Wiesbaden, Germany; growth area 1.12 cm^2^, 0.4 µm pore size) in plating medium (Medium 199 Earle with following supplements: 0.7 mM glutamine, 100 µg/mL penicillin/streptomycine, 100 µg/mL gentamycin, 10% (v/v) newborn calf serum (NCS)) on *day in vitro* (DIV) 2. With the cells seeded on the polycarbonate filters, the apical compartment mimicks the blood and the basolateral compartment the brain side *in vivo*. After two days (DIV 4), PBCEC have reached confluence and medium is changed to serum-free medium (DMEM/Ham's F-12 with 4 mM glutamine, 100 µg/mL penicillin/streptomycine, 100 µg/mL gentamycin) with addition of 550 nM hydrocortisone to trigger differentiation of cells. After two days of differentiation, experiments studying cytotoxicity, barrier integrity and permeability were carried out (DIV 6).

### Studies on Blood-Brain Barrier Integrity

#### 1. Cytotoxicity (CCK-8 assay)

For the determination of general cytotoxic effects of T-2 and HT-2 toxin on PBCEC, the Cell Counting Kit-8 (CCK-8) from Dojindo Laboratories (Tokyo, Japan) was used. The assay was performed as described previously [29]. Briefly, cells were seeded on Collagen-G-coated 96-well plates (250,000 cells/cm^2^) and cultured according to the treatment on Transwell® filter systems (DIV 2: plating medium replaced by serum-free medium on DIV 4 for 2 days). Incubation with various T-2 and HT-2 toxin concentrations (1 nM – 10 µM) was carried out over 24 h and 48 h following the addition of the WST-8 solution [2-(2-methoxy-4-nitrophenyl)-3-(4-nitrophenyl)-5-(2,4-disulfophenyl)-2*H*-tetrazolium, monosodium salt] and incubation according to the manufacturer's manual. Viable cells with active dehydrogenases reduce the tetrazolium salt to a coloured formazan dye, whose absorbance is directly proportional to the number of living cells per well which was measured with a FLUOstar Optima microplate reader (BMG Labtechnologies, Jena, Germany) at 450 nm. For evaluation of viable cells, absorbances of toxin treated cells were compared to those of control cells.

#### 2. TEER Measurement

For monitoring barrier integrity, transendothelial electrical resistance (TEER) was measured with a cellZscope® device (nanoAnalytics, Münster, Germany) during all incubation experiments. A module suitable for 24 Transwell® filter inserts was used. The minimum TEER value for experiments with PBCEC monolayer cultures seeded on Transwell® inserts was 600 Ω*cm^2^. The recorded TEER values for used PBCEC monolayers were averaging between 600 and 1200 Ω*cm^2^.

#### 3. Immunocytochemistry

Immunocytochemical experiments were performed with PBCEC seeded on Transwell® filter inserts as described before. After exposure to different T-2 or HT-2 toxin concentrations for 48 h, cells were fixed in 4% paraformaldehyde (w/v) before application of the primary antibody (1 µg/mL mouse anti-occludin, Zytomed, Berlin, Germany) in 0.5% (w/v) bovine serum albumin (BSA) in phosphate buffered saline (PBS) for 30 min at 37°C. The secondary antibody Alexa Fluor® 546 goat anti-mouse (2 µg/mL, Invitrogen, Paisley, UK) was prepared in 0.5% BSA (w/v) in PBS and incubated as described before. For staining of cell nuclei, filters were further incubated with Hoechst 33258 (bisbenzimide, 10 µg/mL, Sigma-Aldrich) for 30 seconds. Thereafter, filters were cut out from the inserts and placed on coverslips with Aqua Poly/Mount (Polysciences, Washington, USA). Evaluation of immunocytochemical stainging was performed after 24 h of drying with an Axio Imager.M2 (Carl Zeiss, Jena, Germany) fluorescence microscope combined with Axiovision 4.5 software (Carl Zeiss).

#### 4. Western Blot analysis of tight junction protein occluding

For analysis of occludin, PBCEC were grown in Collagen-G-coated 25 cm^2^ culture flasks. Toxin treatment was performed over 24 h following sample preparation according to a previous report [33] with slight modifications. Briefly, cells were washed with PBS and incubated with RIPA-buffer (10 mM Tris, pH 7.6, 150 mM NaCl, 1 mM EDTA, 1% triton-X-100, 1% sodium deoxycholate, 0.1% SDS, 1 µg/mL aprotinin, 1 µg/mL leupeptin, 1 µg/mL pepstatin, 1 mM phenylmethylsulfonylfluorid) for 15 min under shaking on ice. After scraping off, the lysate was sonicated and cell debris was removed by centrifugation (15,000×*g*, 20 min, 4°C). The protein content of the supernatants was quantitated by the Bradford Assay and for each sample 10 µg protein were mixed with loading buffer (Fermentas, St. Leon-Rot, Germany) and denaturated at 95°C for 5 min following electrophoretic separation of proteins by a 12% denaturating sodium dodecylsulfate polyacrylamide gel electrophoresis (SDS-PAGE). For immunoblotting, SDS separated proteins were transferred to a PVDF membrane (Amersham, GE Healthcare, Munich, Germany) by tank blotting. Prior to antibody incubation, the membrane was blocked in Tris buffered saline (TBS) with 0.1% Tween-20 (TBS-T) and 0.5% milk powder (w/v) at room temperature. Then, the membrane was incubated with primary antibodies against occludin or actin (0.5 µg/mL mouse anti-occludin (Zytomed) in 0.5% milk powder in TBS-T; 0.2 µg/mL rabbit anti-actin (Santa Cruz Biotechnology, Heidelberg, Germany) in 5% milk powder in TBS-T) at 4°C overnight. After four times washing in TBS-T for 7.5 min, the membrane was incubated with the horse radish peroxidase conjugated secondary antibodies (0.4 µg/mL goat anti-mouse IgG peroxidase conjugated (Santa Cruz Biotechnology) in 0.5% milk powder in TBS-T; 0.4 µg/mL goat anti-rabbit peroxidase conjugated (Santa Cruz Biotechnology) in 5% milk powder in TBS-T) for 1 h at room temperature. The membrane was washed again four times in TBS-T for 7.5 min a time before visualization of immunoblotting by chemiluminescence using the SuperSignal West Dura kit (Thermo Fisher Scientific, Rockford, USA) according to the manufacturer's instructions in combination with a chemiluminescence imaging system (ChemiDoc XRS, Bio-Rad, Munich, Germany).

#### 5. [^14^C]sucrose Permeability

The application of radiolabeled [^14^C]sucrose to the PBCEC monolayer can additionally serve as a control of barrier tightness besides the measurement of TEER values. Since [^14^C]sucrose is not subjected to transporters or taken up by endothelial cells, it can only permeate to the corresponding compartment in the Transwell® filter system via the paracellular pathway. Therefore, the permeability of [^14^C]sucrose is an appropriate parameter for barrier integrity. After exposure to various concentrations of T-2 or HT-2 toxin respectively, [^14^C]sucrose (Amersham, Buckinghamshire, UK) was applied to the apical compartment and permeability of radiolabeld [^14^C]sucrose was measured to calculate the permeability as described previously [34].

### Studies of barrier integrity, permeability and active transport of T-2 and HT-2 toxin across the BBB in vitro

For studying effects of T-2 and HT-2 toxin on the permeability or active transport across the BBB, the toxins were applied to the apical, basolateral or to both sides simultaneously. A minimum of three to four filters of at least three individual preperations was used including filters exposed to the same amount of solvent (control filters) as present in the toxin treated filters, to exclude any effect of the solvent. Both toxins were applied in a ten-fold concentrated solution in serum-free medium by exchange of 10% of the apical, basolateral or apical and basolateral serum-free medium on DIV 6. Toxin concentrations varied from 25 nM to 75 nM (T-2 toxin) or 50 nM to 200 nM (HT-2 toxin). Barrier integrity was monitored over the whole incubation period by TEER measurements. After different incubation periods (2 h, 24 h, 48 h) experiments were stopped by collecting the whole apical and basolateral medium in separate tubes for sample preparation.

### Quantitation of T-2 and HT-2 toxin

#### 1. Calibration standards

For quantitation of T-2 and HT-2 toxin a QTrap 5500 mass spectrometer (AB SCIEX, Darmstadt, Germany) was coupled to a VWR Hitachi LaChrom Ultra HPLC system (VWR, Darmstadt, Germany). The quantitation of T-2 and HT-2 toxin was performed using a calibration curve with different concentrations of T-2 (0.5 – 15 ng/mL) and HT-2 toxin (1 – 25 ng/mL) in acetonitrile/water (20∶80, v/v). Each calibration point was spiked with 6 ng/mL *d*_3_-T-2 toxin as internal standard.

#### 2. Sample preparation

At different time points after incubation (2 h, 24 h, 48 h), filters were taken out of the cellZscope® device and the whole apical as well as basolateral medium was collected. To each sample, *d*_3_-T-2 toxin (final concentration 6 ng/mL) was added as internal standard before the mixture was evaporated under N_2_ at 37°C. Then, the sample was dissolved to a defined volume with acetonitrile/water (20∶80, v/v), centrifuged (450×*g*, 8 min) and analyzed on a HPLC-MS/MS system. Quantitation was performed by plotting the ratio of the area of the analyte to the area of the internal standard against the analyte concentration.

To investigate any uptake of applied substances by the cells, filters were cut out from the Transwell® inserts and lysed with 1% triton-x 100 solution. Obtained lysates were spiked with internal standard (*d*_3_-T-2 toxin, 6 ng/mL) and analyzed by HPLC-MS/MS as described above.

#### 3. HPLC Parameters

Chromatographic separation was conducted on a 150 mm×2 mm i.d., 4 µm, Synergi Fusion-RP (C18) column with a 4 mm×2 mm i.d. guard column of the same material (Phenomenex, Aschaffenburg, Germany) using a binary gradient with methanol (solvent A) and water (solvent B) both supplemented with 5 mM ammonium acetate. The gradient started at 20% A increasing to 90% A within 7 min, holding these conditions for 1 min before changing to starting conditions at 8.5 min for equilibration, keeping 20% A for an additional 4.5 min resulting in a total run time of 13 min. Flow rate was set to 250 µL/min, injection volume to 10 µL. The first 2.5 min of each run were discarded (divert valve) to remove salts and other polar substances originating from the cell culture medium. Data acquisition was carried out with Analyst 1.5.2 software (AB SCIEX).

#### 4. MS/MS Parameters

For analysis of T-2 and HT-2 toxin, the mass spectrometer was operated in the positive multiple reaction monitoring (MRM) mode. Ion voltage was set to 5500 V with nitrogen serving as collision and curtain gas (35 psi) and zero grade air as nebulizer (35 psi) and drying gas (45 psi) heated to 350°C. Both quadrupoles were set to unit resolution and for all monitored transitions, the declustering potential was set to 21 V, the entrance potential was set to 10 V and collision gas (CAD) was set to “medium”. For quantitation of analytes the ammonium adducts of T-2, HT-2 and *d*_3_-T-2 toxin [M+NH_4_]^+^ were measured with the following transitions and listed collision energy (CE) and cell exit potential (CXP): T-2 toxin, 484 – 185 (CE 27 V, CXP 10 V), 484 – 215 (CE 23 V, CXP 6 V); HT-2 toxin 442 – 215 (CE 17 V, CXP 10 V), 442 – 105 (CE 59 V, CXP 8 V), *d_3_*-T-2 toxin, 487 – 185 (CE 27 V, CXP 10 V), 487 – 215 (CE 23 V, CXP 6 V). Each transition was monitored for 15 ms with the first listed MRM serving as quantifier, the second as qualifier.

### Statistical analysis

Studies of permeability or transport were carried out with at least three different PBCEC monolayers of at least three different preparations (n = 9). Determinations of cytotoxicity using the CCK-8 assay were performed with a minimum of six different wells in three different preparations (n = 18). All results are displayed as mean±SEM. For evaluation of significant differences, the unpaired Student's *t*-test was used with p≤0.05 as statistically significant. The medium effective concentrations (IC_50_ values) were determined on the basis of cytotoxicity data after 48 h toxin incubation with Sigma Plot version 12.0 according to literature [35].

## Results and Discussion

### Cytotoxic effects of T-2 and HT-2 toxin

**Figure 2:**
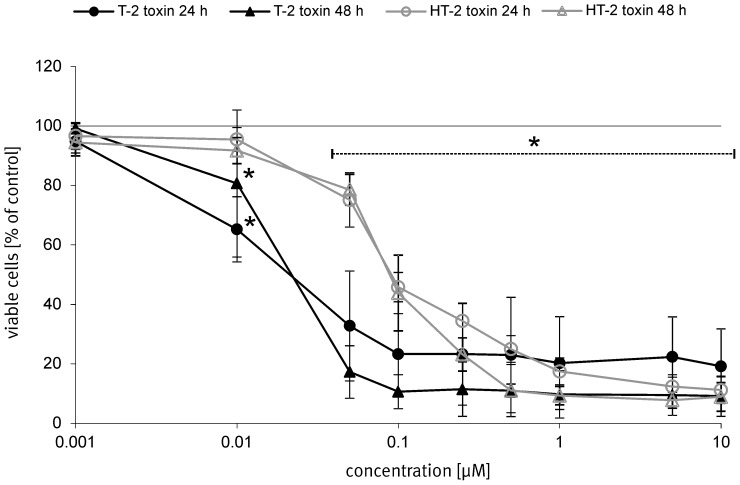
Influence of T-2 and HT-2 toxin on cell viability of PBCEC. Cell viability of PBCEC depending on different T-2 and HT-2 toxin concentrations (1 nM – 10 µM) after 48 h incubation was determined by the CCK-8 assay. Number of analyzed samples *n* = 18 over three individual preparations. Data is given as mean±SEM.

For evaluation of the general cytotoxicity of T-2 and HT-2 toxin on primary porcine brain capillary endothelial cells (PBCEC) mimicking the blood-brain barrier (BBB), the CCK-8 assay was performed. The effects of both trichothecene mycotoxins on PBCEC after application of different concentrations (1 nM – 10 µM) for 24 h and 48 h are displayed in figure 2. Significant (*p*≤0.05) decrease of cell viability to 65% was already observed after incubation of 10 nM T-2 toxin for 24 h whereas incubation of the same concentration HT-2 toxin for the same time period only reduced cell viability to 96%. From 50 nM of both toxins up to the highest incubated concentration of 10 µM, all results regarding reduced cell viability were significant (*p*≤0.05) compared to solvent treated control cells. After an extended incubation time of 48 h, cell viability curves were similar to those reported for T-2 and HT-2 toxin after 24 h respectively: cell viability was reduced to 17% (T-2 toxin) or 79% (HT-2 toxin) at concentrations of 50 nM T-2 or HT-2 toxin after 48 h incubation. With higher concentrations, cytotoxic effects of both toxins became more pronounced: cell viability of HT-2 toxin incubated cells decreased in a concentration dependent manner to a minimum of about 10% at concentrations of 500 nM. For T-2 toxin, cell viability was reduced to about 10% after incubation of 100 nM and was not reduced to a much lower percentage at higher incubation concentrations. The IC_50_ value for T-2 toxin was calculated with 18 nM(±0.6 nM). For HT-2 toxin the IC_50_ value was evaluated with 84 nM(±6.1 nM). These values are remarkable since primary cells of human origin (human renal proximal tubule epithelial cells, RPTEC) showed an about 12-fold higher IC_50_ value after T-2 toxin incubation (200 nM) and a 9-fold higher value for HT-2 toxin [10] indicating that PBCEC react very sensitive against T-2 and HT-2 toxin. The different origin of the used primary cells might be responsible for the difference in sensitivity to the tested trichothecenes. Primary human astrocytes though, showed a similar IC_50_ value after T-2 toxin incubation (24 nM) [24] indicating a high susceptibility of cerebral cells against T-2 toxin. The difference in the cytotoxic reaction of PBCEC to T-2 or HT-2 toxin respectively is further interesting as cell viabilities decreased to a different extent after incubation of 50 nM (T-2 toxin: 17%; HT-2 toxin: 78%) but a similar reduction of cell viability was observed at higher concentrations (starting at 250 nM) for both toxins (viability reduced to 10%).

### TEER measurements after T-2 and HT-2 toxin application

#### 1. Effects of T-2 toxin

**Figure 3:**
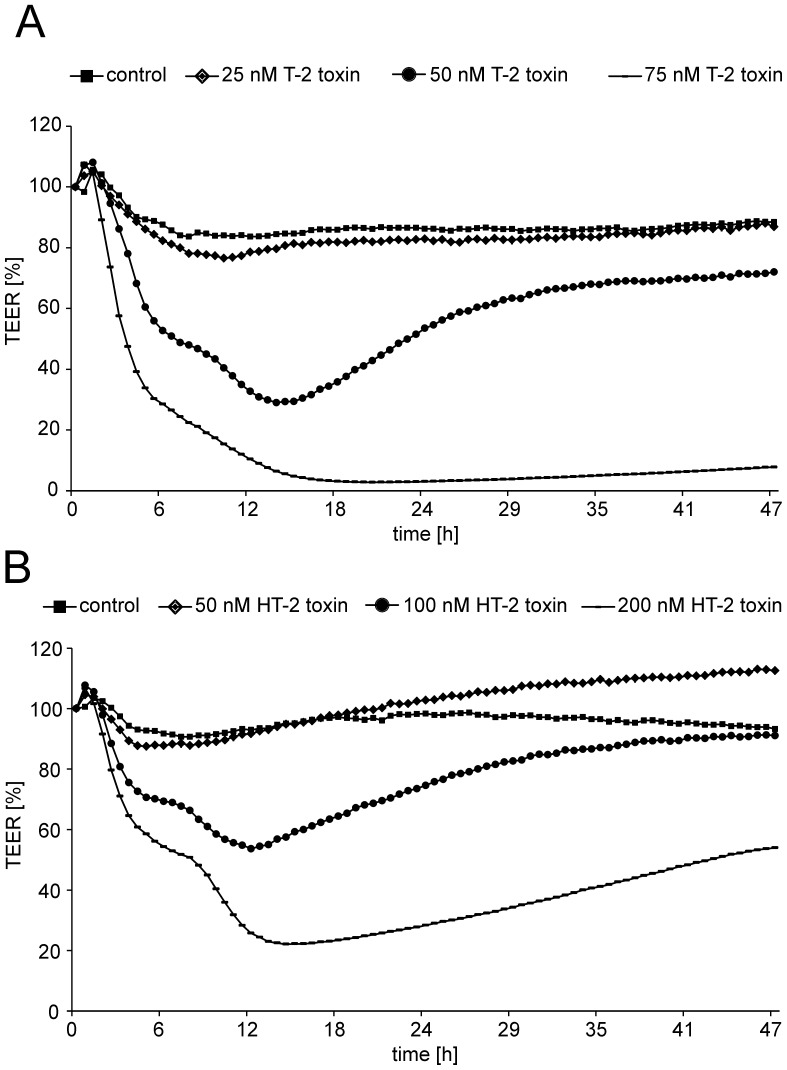
Effects of T-2 and HT-2 toxin on barrier function of PBCEC over 48 h. A: TEER measurements after incubation of various concentrations of T-2 toxin from the apical side to PBCEC monolayers. B: Results of TEER measurements after incubation of different HT-2 toxin concentrations from the apical side to PBCEC monolayers. all: n = 9 with standard deviations 10 – 15% (not shown).

**Figure 4:**
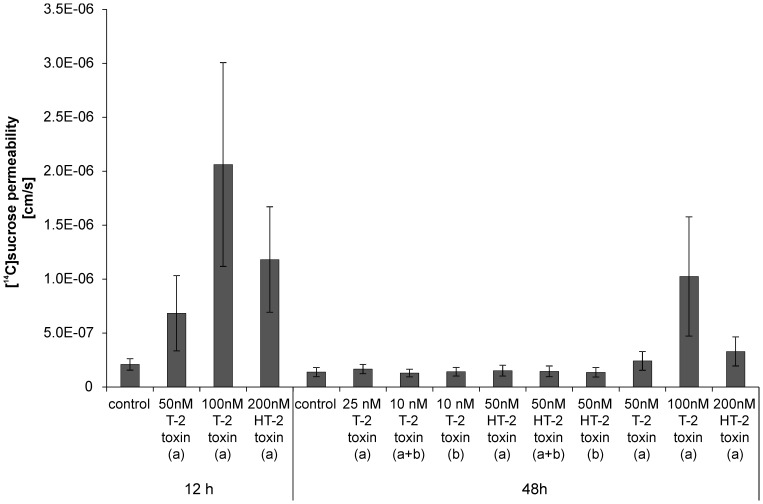
Barrier integrity after toxin incubation as determined by [^14^C]sucrose permeation. Permeation of [^14^C]sucrose was used as an additional indicator for barrier integrity after incubation of different concentrations of T-2 and HT-2 toxin for 12 h and 48 h from the apical side (a), the basolateral side (b) or from both sides (a+b); all: mean±SEM; n = 3.

The impact of T-2 and HT-2 toxin on the barrier integrity was analyzed by transendothelial electrical resistance (TEER) measurements during toxin incubation and additionally by [^14^C]sucrose permeability determination. Changes in barrier integrity are displayed in figure 3 in percentage of starting TEER values. For T-2 toxin applied from the apical side (Fig. 3 A), concentrations of 50 nM yielded in a decreased barrier integrity already after 6 h (50% of initial TEER value) and continued to a minimum of about 30% of initial TEER value after 14 h to 15 h incubation. Interestingly, PBCEC seem to be able to restore barrier functions as the TEER value starts to increase from 15 h on, resulting in a plateau of about 60% of the initial TEER value at 30 h of incubation keeping this TEER until 48 h. To verify these results, [^14^C]sucrose permeability was additionally measured (Fig. 4) after incubation of 50 nM T-2 toxin. These experiments revealed comparable results with control cells showing a [^14^C]sucrose permeability of 2.10±0.53×10^−7^ cm/s at 12 h while 50 nM T-2 toxin incubated cells exhibited a higher permeability of 6.84±3.49×10^−7^ cm/s after 12 h incubation. At an extended incubation period of 48 h, control cells had a permeability of 1.38±0.43×10^−7^ cm/s which is comparable to the one after 12 h. Incubation with 50 nM T-2 toxin for 48 h though, resulted in a [^14^C]sucrose permeability of 2.42±0.88×10^−7^ cm/s which is lower than that one measured after 12 h incubation and in the same range like control cells, indicating the restoration of barrier function after prolonged incubation of T-2 toxin. At higher concentrations barrier function was lost without recovery: incubation of 75 nM T-2 toxin lead to a rapid decrease of TEER values (30% of initial TEER 6 h past incubation) until less than 10% of initial TEER were measured (<50 Ω*cm^2^) at 18 h after toxin application. This plateau of low TEER values did not change up to the end of incubation experiments at 48 h.

Similar results were obtained after incubation of 100 nM T-2 toxin from the apical side (data not shown). [^14^C]sucrose experiments confirmed these results and incubation with 100 nM T-2 toxin (48 h) resulted in a permeability of 1.02±0.55×10^−6^ cm/s. Incubations with 25 nM T-2 toxin however, did not affect the barrier integrity over 48 h ([^14^C]sucrose permeability (48 h): 1.66±0.44×10^−7^ cm/s). Thus, 25 nM T-2 toxin applied from the apical side were chosen for permeability studies to guarantee intact barrier function.

When applied from the basolateral side, referring to the brain side *in vivo*, PBCEC were more sensitive against T-2 toxin resulting in barrier impairment at concentrations of 25 nM T-2 toxin (data not shown). Therefore, 10 nM T-2 toxin were chosen for permeability studies of T-2 toxin from the basolateral side maintaining a barrier function ([^14^C]sucrose permeability: 1.42±0.40×10^−7^ cm/s) in the same range as control cells over 48 h.

The same concentration was chosen for studies of active transport where the toxin was applied from both sides (apical and basolateral). The addition of equimolar concentrations in both compartments of the Transwell® filter insert indicates an active transport when an enrichment in one specific compartment is detectable. Active transport experiments could not be performed with 25 nM T-2 toxin applied from both sides since this concentration caused barrier disruption (data not shown). However, the barrier stayed intact over 48 h after exposure to 10 nM T-2 toxin applied from the apical and basolateral side with a [^14^C]sucrose permeability of 1.30±0.35×10^−7^ cm/s which is comparable to control cells (Fig. 4).

#### 2. *Effects of HT-2 toxin*

HT-2 toxin did not impair barrier function in the same concentration ranges as T-2 toxin. After application of 100 nM HT-2 toxin from the apical side (Fig. 3 B), TEER decreased to about 70% of initial value after 6 h of incubation resulting in a minimum of about 55% of the initial TEER after prolonged incubation of 12 h. However, from 12 h on, TEER values recovered resulting in values in the same range as those of control cells after 48 h incubation. Regeneration of TEER values has already been described for other substances in the literature [32]. Doubling of the concentration to 200 nM HT-2 toxin decreased barrier integrity to about 20% after 12 h incubation (Fig. 3 B). Again, TEER values increased over the following hours leading to 40% of the initial TEER value at 48 h after incubation. This effect was verified by [^14^C]sucrose permeability with 1.18±0.5×10^−6^ cm/s at 12 h after incubation of 200 nM HT-2 toxin, decreasing to 3.23±1.35×10^−7^ cm/s after 48 h incubation (*cf.* control at 48 h: 1.38±0.43×10^−7^ cm/s). A concentration of 50 nM HT-2 toxin was chosen for permeability studies since no barrier impairment was detectable (*cf*. [^14^C]sucrose permeability 1.52±0.50×10^−7^ cm/s).

For the application of HT-2 toxin to the basolateral or to both sides (apical and basolateral), the same concentration as used for apical exposure was applied. In both cases no change in TEER values was observed (data not shown). [^14^C]sucrose permeabilities were in the same ranges as for control cells: 50 nM HT-2 toxin, basolateral, 48 h: 1.37±0.44×10^−7^ cm/s; 50 nM HT-2 toxin, apical and basolateral, 48 h: 1.45±0.50×10^−7^ cm/s.

A strong difference in toxic response and concentration dependency was seen between T-2 and HT-2 toxin. While concentrations of 75 nM T-2 toxin applied from the apical side resulted in total barrier disruption, concentrations up to 100 nM HT-2 toxin were tolerable for PBCEC and barrier function could be restored to a certain extent whereas TEER values did not recover over 48 h after 75 nM T-2 toxin application.

#### Effects on tight junction protein occludin

**Figure 5:**
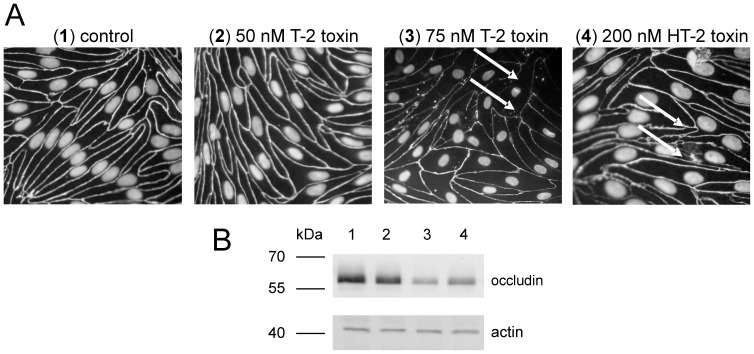
Immunocytochemical staining of occludin after T-2 and HT-2 toxin incubation from the apical side. A: Immunocytochemical staining of tight junction protein occludin in PBCEC after T-2 or HT-2 toxin incubation for 48 h from the apical side. TEER of control (A, 1) and 50 nM T-2 toxin (A, 2) incubated cells>1000 Ω*cm^2^; TEER of 75 nM T-2 toxin (A, 3) incubated filters>50 Ω*cm^2^; TEER of 200 nM HT-2 toxin (A, 4) incubated filters>600 Ω*cm^2^. Regions of lost or impaired occludin are indicated by arrows. B: Western blotting of occludin with actin as loading control of control filters (B, 1), 50 nM T-2 toxin (B, 2), 75 nM T-2 toxin (B, 3) and 200 nM HT-2 toxin (B, 4) incubated PBCEC for 24 h.

To investigate the effects of T-2 and HT-2 toxin on barrier characteristics like the tight junction protein occludin, fluorescence microscopy after immunocytochemical staining was performed after incubation of T-2 and HT-2 toxin for 48 h (Fig. 5 A). While control cells show clear and regular lines for occludin (Fig. 5 A (1)), toxin incubated cells show a different pattern: incubation of 75 nM T-2 toxin from the apical side (Fig. 5 A (3)) results in disturbed and irregular occludin staining including holes in the occludin distribution. At smaller concentrations of 50 nM T-2 toxin (Fig. 5 A (2)), occludin tightness is not changed in comparison to control cells. These findings correspond well to observed changes in TEER values where barrier function was retained after incubation of 50 nM T-2 toxin for 48 h.

Additionally, incubation with 200 nM HT-2 toxin for 48 h from the apical side lead to more irregular occludin borders which are different from control cells (Fig. 5 A (4)). Albeit clear differences in occludin protein were observed between control and T-2 toxin incubated cells (75 nM), the effects after HT-2 toxin exposure (200 nM) were not as distinct after immunocytochemical staining. Thus, western blot analysis of occludin was performed after 24 h of toxin incubation. Figure 5 B shows the results of immune blotting of occludin, and actin as loading control. The results from fluorescence microscopic studies were confirmed for the incubated toxins with 50 nM T-2 toxin (Fig. 5 B, lane 2) not decreasing occludin in comparison to control cells (Fig. 5 B, lane 1), whereas incubation of 75 nM T-2 toxin (Fig. 5 B, lane 3) lead to a decreased signal for occludin. Moreover, application of 200 nM HT-2 toxin (Fig. 5 B, lane 4) yielded in reduced occludin levels in comparison to control cells. Therefore immunocytochemical staining of occludin after incubation with 200 nM HT-2 toxin was confirmed by western blot analysis. The observed loss of the tight junction protein occludin might be responsible for the lower barrier integrity as tight junctions form the physical barrier of the BBB. Again, a difference in concentration dependent effects was observed for T-2 and HT- toxin (reduction of occludin: 75 nM (T-2 toxin); 200 nM (HT-2 toxin)).

#### *Permeability of T-2 and HT-2 toxin*

**Figure 6:**
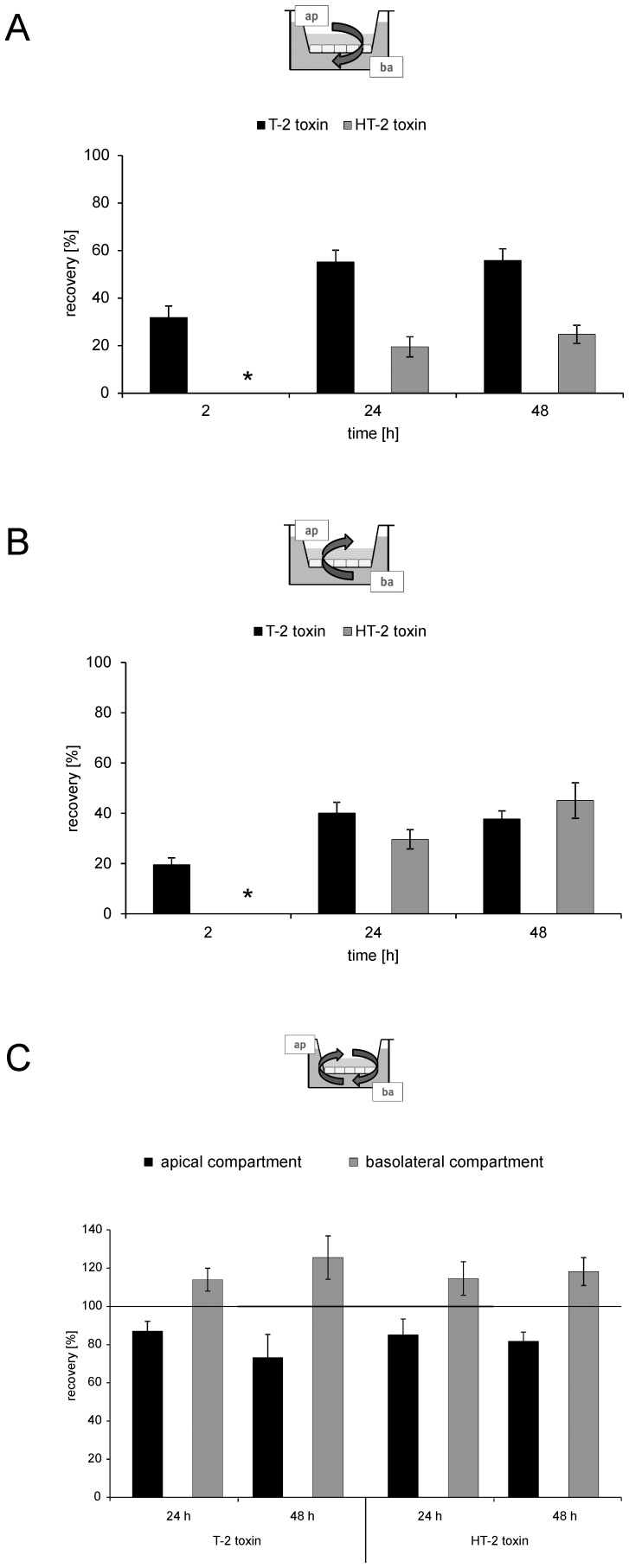
Permeation of T-2 and HT-2 toxin across PBCEC monolayers after various time points. A: Recovery of T-2 and HT-2 toxin (% of initially applied toxin) in the basolateral compartment after application of toxin (T-2 toxin: 25 nM; HT-2 toxin: 50 nM) to the apical compartment for 2 h, 24 h and 48 h. B: Recovery of T-2 and HT-2 toxin (% of initially applied toxin) in the apical compartment after application of toxin (T-2 toxin: 10 nM; HT-2 toxin: 50 nM) to the basolateral compartment for 2 h, 24 h and 48 h. C: Recovery of T-2 and HT-2 toxin (% of initially applied toxin) in the apical and basolateral compartment after application of equimolar concentrations of T-2 (10 nM) and HT-2 (50 nM) toxin to both compartments (apical and basolateral) for 24 h and 48 h.* indicates only traces detected; all: mean±SEM with a minimum of n = 6.

To evaluate permeation of T-2 and HT-2 toxin across the BBB *in vitro* toxins were either applied from the apical or the basolateral side. Figure 6 A displays the recovery of toxins in the corresponding basolateral compartment after application of 25 nM T-2 toxin or 50 nM HT-2 toxin respectively, from the apical side in a time dependent manner. After 2 h, 32%(±5%) of applied T-2 toxin were detected in the basolateral compartment resembling the brain side *in vivo*. After the same incubation period, HT-2 toxin was not detectable in the basolateral segment. After longer incubation periods, T-2 toxin permeation increased but seemed to reach saturation after 24 h with 55%(±5%) and 56%(±5%) after 48 h. The same tendency was observed for HT-2 toxin permeation but lower amounts were detected in the basolateral compartment compared to T-2 toxin (20%±4% after 24 h; 25%±4% after 48 h). The difference in permeation between T-2 and HT-2 toxin is visible at all tested incubation times revealing a fast permeation of T-2 toxin across the BBB from the mimicked blood to the brain side with an accumulation of T-2 toxin on the basolateral side.

This hypothesis becomes even more relevant when applying T-2 and HT-2 toxin from the basolateral side (T-2 toxin: 10 nM; HT-2 toxin: 50 nM). Again, a fast permeation of T-2 toxin is visible with 20%(±3%) of the initially applied toxin present in the corresponding apical compartment after 2 h (Fig. 6 B). After 24 h and 48 h, permeation rates for T-2 toxin are similar with 40%±4% (24 h) and 38%±3% (48 h) but are again lower than for the application from the apical side. However, a comparison of absolute recovery rates between application of T-2 toxin from the apical (Fig. 6 A) or the basolateral side (Fig. 6 B) respectively, shows that the permeation rate from the apical (blood) to the basolateral (brain) side is higher than vice versa indicating an accumulation of T-2 toxin on the basolateral side.

A different picture was observed when focusing on the permeation of HT-2 toxin from the basolateral to the apical side (Fig. 6 B): after 24 h, about 30%(±4%) of applied toxin are detectable on the apical side with an increase to 45%(±7%) after 48 h, which is higher than for T-2 toxin after 48 h (*cf.* 38%±3%). Both permeation values after 24 h and 48 h are higher than for the application from the apical side (Fig. 6 A) (*cf.* 20%±4% (24 h); 25%±4% (48 h)) suggesting a preferred permeation from the brain (basolateral) to the blood (apical) side for HT-2 toxin. In general, the permeation of T-2 and HT-2 toxin from the basolateral to the apical compartment is similar after 48 h, however no detectable permeation was seen for HT-2 toxin after 2 h.

The permeation values for T-2 and HT-2 toxin were estimated based on permeation data after 48 h with the following values for the permeation from the apical to the basolateral side: T-2 toxin, 5.72±0.50×10^-6^ cm/s; HT-2 toxin 2.53±0.39×10^−6^ cm/s. These values are in a low range compared to the permeability of ergot alkaloid mycotoxins reported earlier using the same model system which showed higher permeation from the apical to the basolateral side [29]. For permeation from the basolateral to the apical side, the value for T-2 toxin was 1.94±0.16×10^−6^ cm/s and for HT-2 toxin 2.31±0.36×10^−6^ cm/s. The permeation of morphine (*cf*. 1.6±0.3×10^−6^ cm/s) was also studied on the used cell type monolayer and exhibited permeation in a comparable range as T-2 and HT-2 toxin [36].

In the here presented study, the distribution of T-2 and HT-2 toxin was additionally analyzed in PBCEC after lysis of the cells grown on the filter inserts. After all tested conditions (application from apical, basolateral or both sides; different incubation periods) no toxin was detectable in the lysate of PBCEC indicating that both toxins are not accumulated inside the cells. These results correspond well to the recovery rates of toxin applied to the apical or basolateral compartment summing up to 100% in total (data not shown). Nevertheless, in comparison to literature data, these findings are interesting as the application of ergot alkaloid mycotoxins on PBCEC showed an uptake of substances (ergotamine, ergocristinine) of up to 60% in the cell lysate [29]. However, since permeation across the BBB was detected for T-2 and HT-2 toxin in this study, but barrier integrity was proven for the tested concentrations by TEER and [^14^C]sucrose permeability measurements, both toxins seem to cross the barrier via the transcellular route as the BBB forms a physical barrier with intact tight junctions. However, the permeation is likely to occur fast, because no toxins were detected in the cell lysates. Further studies should focus on specific transporters being involved in T-2 or HT-2 toxin permeation across the BBB.

Concerning the question if the toxins are subjected to an active transport across the BBB, only weak indications were found in this study after application of equimolar concentrations of T-2 and HT-2 toxin from both sides (T-2 toxin: 10 nM; HT-2 toxin: 50 nM). Enrichment of applied toxin was observed for both toxins in the basolateral compartment after 24 h and 48 h with concurrent decrease of toxin concentration on the apical side (Fig. 6 C). Despite differences between the two toxins when applied from the apical or basolateral side, a similar tendency after application from both sides was observed with a slight elevation (about 115%) of T-2 and HT-2 toxin in the basolateral compartment indicating an active transport.

Another noteworthy aspect is the lacking metabolism of T-2 toxin to HT-2 toxin in PBCEC. No HT-2 toxin was detectable after incubations with T-2 toxin, while this conversion is well described in literature for various cell types *in vitro*: primary cells of different human origin like RPTEC (kidney), NHA (astrocytes), NHLF (lung fibroblasts) as well as cell lines derived from human colon carcinoma cells (HT-29) metabolize T-2 toxin rapidly to HT-2 toxin [10], [11]. Metabolism studies mimicking the human gastrointestinal tract found also HT-2 toxin as the main or even sole metabolite after T-2 toxin application [25]. In PBCEC, the lack of metabolizing T-2 toxin could also be partly responsible for the high sensitivity of these cells against T-2 toxin. For general cytotoxicity, the calculated IC_50_ value after HT-2 toxin incubation was about 4 times higher than that of T-2 toxin and also differences in concentration dependent disruption of barrier function as indicated by TEER measurements were detected in PBCEC in this study. Since HT-2 toxin exhibits a slightly lower cytotoxicity on PBCEC, however the cells are not able to convert T-2 toxin to HT-2 toxin, toxic effects after T-2 toxin application could be more severe than for HT-2 toxin.

Overall, T-2 and HT-2 toxin show strong effects on the BBB *in vitro*. Cytotoxicity was evaluated for both toxins with IC_50_ values in the low nanomolar range. Total disruption of barrier function was detected at 75 nM T-2 toxin and at 200 nM HT-2 toxin with slight recovery in the case of HT-2 toxin. Moreover, the two tested mycotoxins are able to penetrate across the BBB as mimicked by PBCEC in this study. Besides the fast permeation of T-2 toxin already after 2 h, permeation of HT-2 toxin occurred after longer incubation times. As loss of tight junction protein occludin was visible after T-2 and HT-2 toxin incubations, barrier disruption can be explained. In general the toxic effects on the BBB *in vitro* were detected at low nanomolar concentrations. However, when comparing the results to the situation *in vivo*, it has to be taken into account that the used model system does not include serum which can modify the availability of T-2 and HT-2 toxin under *in vivo* conditions.

The results of the present study show impairment of the BBB *in vitro*, an aspect of T-2 toxin which has until now only been considered sparsely. To our knowledge effects of HT-2 toxin on the CNS were until now not studied in detail. With strong cytotoxic effects at low nanomolar concentrations, neurotoxic properties should be further investigated for the two common contaminants of food and feed commodities.
